# Post and Core Restorations of Endodontically Treated Teeth: A Cross-Sectional Study of Moroccan Dentists

**DOI:** 10.7759/cureus.111224

**Published:** 2026-06-21

**Authors:** Sofia Drouri, Hanane Tantanat, Yassine El Ghachi, Mouna Hamza, Mouna Jabri

**Affiliations:** 1 Department of Conservative Dentistry and Endodontics, Faculty of Dental Medicine, Hassan II University, Casablanca, MAR; 2 Department of Pediatric Dentistry, Community Health, Epidemiology and Biostatistics Laboratory, Faculty of Dental Medicine, Hassan II University, Casablanca, MAR

**Keywords:** direct techniques, endodontically treated teeth, fiber post, indirect techniques, post and core

## Abstract

Background: The loss of tooth structure following endodontic treatment weakens endodontically treated teeth, often requiring post and core to ensure the stability of restorations. Post and core restoration can be performed using direct techniques, which involve a prefabricated post combined with a coronal reconstruction in a single session, or using indirect techniques requiring the fabrication of an inlay core in a laboratory.

Aim and objectives: This study aimed to evaluate the techniques and materials currently used by dentists in the Casablanca-Anfa prefecture.

Materials and methods: A descriptive cross-sectional study was conducted between February and June 2024 among private dental practitioners in the Casablanca-Anfa prefecture, Morocco. A simple random sample of 245 dentists was selected from the official registry of the Southern Regional Council of Dentists. Data were collected using a validated structured questionnaire assessing practitioners’ demographics and corono-radicular restoration practices. Statistical analysis was performed using SPSS version 20.0, with qualitative variables expressed as frequencies and percentages. A total of 244 valid responses were analyzed.

Results: Direct post and core restorations were frequently used in 90.2% of cases, mainly with fiberglass posts (91.5%), non-adhesive cements (48.5%), and flowable composite (73.3%). Indirect techniques were reserved for severe decay, and computer-aided design/computer-aided manufacturing (CAD/CAM) inlay cores were rarely used (5.2%).

## Introduction

The endodontically treated tooth undergoes several modifications related to endodontic treatment, including a decrease in proprioception, variations in its water content, and mechanical and histological alterations. While the physical and chemical effects of endodontic treatment have a relatively minor influence on the long-term prognosis of the tooth, it is mainly the loss of tooth substance that weakens the tooth structure and impacts its prognosis. Indeed, a study conducted by Reeh and colleagues in 1989 showed that tooth strength decreases in proportion to the amount of tissue lost [1].

Indeed, after removing decayed tissue and performing endodontic treatment, the tooth either has no walls or has weakened residual walls, making it impossible to perform a direct restoration that will last in the long term. An endodontic post then becomes essential to ensure the stability and durability of the crown restoration [2]. Two therapeutic approaches are available for post and core restorations: direct techniques using a prefabricated post and indirect techniques based on a laboratory-fabricated inlay core [3]. The choice of technique and materials depends on clinical factors, practitioner preferences, training, and socioeconomic context. An evaluation of current practices is therefore essential to better understand clinical trends [4].

This study aimed to determine the techniques and materials most commonly used by dentists in the Casablanca-Anfa prefecture, Morocco, for post and core restorations.

## Materials and methods

This research was designed as a descriptive cross-sectional epidemiological study. The investigation was conducted over a five-month period, from February to June 2024. The study setting comprised private dental practices and centers located within the Casablanca-Anfa prefecture in Casablanca, Morocco. The study population consisted Fof dental surgeons practicing in the private sector.

Inclusion criteria

To be eligible, practitioners had to be registered with the Southern Regional Council, maintain an active practice within the Casablanca-Anfa prefecture, perform prosthetic dental care, and provide informed consent to participate.

Exclusion criteria

The study excluded specialists whose primary focus did not include prosthetic restorations, specifically orthodontists, periodontists, and oral surgeons exclusively engaged in their respective specialties. Practitioners located outside the defined geographical boundaries of Casablanca-Anfa were also excluded.

The sampling frame was derived from the official registry provided by the Southern Regional Council of Dentists via email, which listed all 629 private-sector practitioners in Casablanca-Anfa. From this total population, a simple random sampling method was employed to select the participants. The sample size was statistically calculated and established at 245 practitioners from a total of 629 registered dentists.

A structured, five-page questionnaire in French served as the primary data collection tool. It comprised 29 questions categorized into four thematic sections: (1) Sociodemographic Characteristics: including age, gender, years of experience, and place of graduation; (2) General CRR Practices: knowledge and general clinical habits regarding corono-radicular restoration (CRR) of endodontically treated teeth; (3) Direct CRR Techniques: specifics on the use of prefabricated posts and direct restoration materials; and (4) Indirect CRR Techniques: focus on cast post-and-core (inlay-core) procedures (Appendices).

The questionnaire was developed based on a review of the relevant literature and was reviewed by the thesis director, a professor in Conservative Dentistry and Endodontics at the Faculty of Dental Medicine of Casablanca, who assessed its content validity prior to the pilot study.

To ensure the instrument's face validity, clarity, and acceptability, a pilot study was conducted among 10 randomly selected practitioners from the private sector in the Casablanca-Anfa prefecture. This phase confirmed that the questionnaire was easily understood and required approximately five minutes to complete. As no difficulties were identified during this pilot phase, the questionnaire was retained without further modification and considered valid for use in the main survey.

The validated questionnaires were distributed manually by the researcher through direct visits to each of the selected dental practices. This personalized approach contributed to an exceptionally high participation rate of 99.59%, with 244 of 245 practitioners providing valid responses.

Raw data were initially entered into a Microsoft Excel spreadsheet and subsequently exported for statistical processing using IBM SPSS Statistics for Windows, Version 20 (Released 2011; IBM Corp., Armonk, New York). The analysis was performed at the Community Health Laboratory of the Faculty of Dental Medicine in Casablanca. A descriptive analysis was used for all study parameters, with qualitative variables expressed as absolute frequencies (N) and percentages (%).

## Results

The study included a total of 245 dentists, 244 of whom agreed to respond to our questionnaire, representing a participation rate of 99.59%. The study population was predominantly female (60.7%). There was a predominance of practitioners under the age of 40, with 28.3% of participants aged 30-39 years. In addition, 29.9% of dentists had been practicing for more than 20 years. In terms of education, 82% of participants were graduates of Moroccan universities. The general characteristics of the participants are presented in Table 1. 

**Table 1:** General information of dentists (N = 244).

Variables	n (%)
Gender	
Female	148 (60.7)
Male	96 (39.3)
Age groups	
20-30 years	68 (27.9)
30-40 years	69 (28.3)
40-50 years	45 (18.4)
50-60 years	41 (16.8)
≥60 years	21 (8.6)
Year of practice	
<5 years	63 (25.8)
5-10 years	40 (16.4)
10-20 years	68 (27.9)
≥20 years	73 (29.9)
Location of degree obtained	
Morocco	200 (82)
Abroad	44 (18)

Table 2 summarizes the participants’ responses regarding post and core restoration practices.

**Table 2:** Questionnaire overview with results. CAD/CAM: computer-aided design/computer-aided manufacturing.

Questions	Options	n (%)
Routine performance of post and core restorations	Yes	242 (99.2)
	No	2 (0.8)
Belief that every endodontically treated tooth systematically requires a post	Yes	209 (85.7)
	No	35 (14.3)
Timing of post space preparation	Immediately after endodontic treatment	150 (61.7)
	24 hours after endodontic treatment	55 (22.6)
	One week after endodontic treatment	49 (20.2)
	A few weeks after endodontic treatment	25 (10.3)
Rubber dam usage during post and core restoration	Never	183 (75)
	Sometimes	57 (23.4)
	Always	4 (1.6)
Apical gutta-percha remnant height	4-5 mm	131 (53.7)
	3 mm	82 (33.6)
	Other	20 (8.2)
	2 mm	11 (4.5)
Gutta-percha removal method (Multiple choices allowed)	Gates Glidden	218 (89.3)
	Without solvent	73 (29.9)
	Root canal retreatment files	24 (9.8)
	Manual files	16 (6.6)
	With gutta-percha solvents	16 (6.6)
Final canal irrigation solution	Chlorhexidine	124 (50.8)
	EDTA (ethylene diamine tetra-acetic acid) solution	106 (43.4)
	Sodium hypochlorite	45 (18.4)
	I do not rinse the canal	10 (4.1)
Belief that posts structurally strengthen the root	Yes	214 (88)
	No	15 (6.2)
	I do not know	14 (5.8)
Belief that the ferrule effect increases fracture resistance	Yes	140 (58.3)
	No	96 (40)
	I do not know	4 (1.7)
Performance of direct post and core restorations	Yes	220 (90.2)
	No	24 (9.8)
Anatomical sector for direct post and core restoration (Multiple choices allowed)	Anterior teeth	204 (92.7)
	Posterior teeth	154 (70)
Indications for direct post and core restoration selection (Multiple choices allowed)	Two persistent residual walls	140 (63.9)
Type of prefabricated post utilized (Multiple choices allowed)	Fibers posts	213 (94.7)
	Glass fiber	205 (91.5)
	Quartz fiber	4 (1.8)
	Carbon fiber	10 (4.5)
	Metal posts	109 (48.4)
	Screw post	75 (33.5)
	Titanium tenons	12 (5.4)
	Stainless steel tenons	27 (12.1)
	Ceramic posts	30 (13.3)
Luting/bonding systems for posts	The adhesive used	
	Dual-cure adhesives	121 (53.3)
	Nonadhesive cement	110 (48.5)
	Self-adhesive cement	49 (21.6)
	Self-cure adhesive	30 (13.2)
	Light-cure adhesive	16 (7)
Adhesive strategy if using nonadhesive cements	Bonding systems	
	Self-etching systems	94 (48.7)
	Universal adhesives	77 (39.9)
	Etch and rinse adhesives	30 (15.5)
Coronal core build-up material	Coronary restoration materials	
	Composite	205 (92.3)
	Flowable composite	148 (73.3)
	Packable composite	76 (37.6)
	Composite + glass ionomer cement	15 (6.8)
Performance of indirect cast post restorations	Yes	187 (77.6)
	No	54 (22.4)
Anatomical sector for indirect cast posts	Anterior teeth	185 (96.4)
	Posterior teeth	99 (51.5)
Clinical scenarios for indirect cast posts (Multiple choices allowed)	Significant decay with a marginal line at or slightly below the gingival margin	176 (92.1)
	Insufficient residual walls to consider a crown restoration	130 (68.1)
	Root canal anatomy is incompatible with a prefabricated post	32 (16.8)
Type of inlay-core material used	Metal inlay core	181 (94.3)
	Ceramic inlay core	38 (19.8)
	CAD/CAM fiber inlay core	10 (5.2)
	Other	2 (1)
Luting material for indirect inlay-cores	Glass ionomer cement	174 (91.6)
	Zinc oxyphosphate cement	13 (6.8)
	Other	6 (3.1)
	Zinc polycarboxylate cement	3 (1.6)

Most dentists reported performing post and core restorations on endodontically treated teeth (99.2%). In addition, 85.7% believed that endodontically treated teeth automatically require the placement of a post. Concerning clinical practices, 61.7% performed post and core restorations immediately after endodontic treatment, whereas only 1.6% systematically used a rubber dam during the procedure. Regarding post space preparation, 53.7% reported leaving 4-5 mm of gutta-percha apically.

Fiber posts were the most commonly used prefabricated posts (94.7%), particularly glass fiber posts (91.5%). Composite resin was the preferred material for direct post and core restorations (92.3%). Furthermore, 77.6% of practitioners reported performing cast post and core restorations, mainly in cases of extensive coronal destruction (92.1%).

## Discussion

Advances in adhesive dentistry have enabled more conservative approaches to restoring endodontically treated teeth. Alternatives such as direct restorations and indirect partial restorations (inlays, onlays, overlays, and endocrowns) are now preferred before considering post-and-core restorations [5].

In the present study, 61.7% of practitioners perform root canal preparation for the post one week after endodontic treatment, a rate close to the 64.66% reported in India [6]. Immediate root canal preparation after root canal treatment would allow for better apical sealing and reduce the risk of bacterial infiltration [7]. This is especially true if the preparation is performed using a dental dam, which is optimal for material adhesion. In this study, 75% of practitioners do not use a dental dam, a rate comparable to that reported in Brazil [8]. However, its use contributes significantly to the success and durability of post and core restorations [9].

Also, 53.7% of practitioners leave 4 to 5 mm of gutta-percha during preparation for a root canal post. This result is comparable to a study in India, where 88.47% of practitioners do the same [6]. In contrast, 33.6% of practitioners left only 3 mm.

The preferred method is based on the use of Gates Glidden drills without solvent, also reported in the literature. This approach, through mechanical removal, reduces the formation of dentinal sludge and facilitates the cleaning of canal walls [10].

With regard to peripheral cervical preparation, the ferrule effect is a key factor in preventing tooth fractures. Its presence ensures even stress distribution and significantly reduces the risk of failure, even exceeding the importance of using a post [11].

Final irrigation and post and core restoration

In this study, 2% chlorhexidine and 2.5% sodium hypochlorite were the most commonly used solutions for final irrigation prior to sealing or bonding the post, with respective percentages of 50.8% and 43.4%, while 18.4% of practitioners did not perform this step. In an Indian study, 82.45% of practitioners preferred sodium hypochlorite as their final irrigation solution [6].

Final irrigation aims to remove canal debris and cement residues that could compromise the adhesion of the post to the root dentin [12]. A 17% EDTA solution is recognized as the most effective for removing dentinal sludge. The recommended sequence consists of an initial rinse with sodium hypochlorite, followed by activation with 17% EDTA, then a final rinse with 2% chlorhexidine [13]. The absence of irrigation can alter the quality of the post/dentin bond, regardless of the material used, thereby compromising the long-term stability of post and core restorations [14].

Types of posts for post and core restorations

Metal inlay cores remain widely used for cast post and core restorations due to their mechanical strength and adaptability to irregular canals. However, their rigidity, which is greater than that of dentin, can generate internal stresses that increase the risk of root fractures [3]. Their use in the posterior region remains common for functional reasons, but esthetic concerns are increasingly leading to the use of ceramic inlay cores. Computer-aided design/computer-aided manufacturing (CAD/CAM) inlay cores, although not yet widely used, offer high precision, increased strength, and excellent esthetics [15].

Prefabricated fiberglass posts are the most commonly used (91.5%) in this study. Their modulus of elasticity, which is similar to that of dentin, promotes even stress distribution, reducing the risk of fractures, and their esthetic properties make them ideal for anterior teeth [16]. Quartz fiber posts offer similar advantages, with excellent optical integration and the potential for repair in the event of fracture [17].

The innovative fiberglass "FiberSite Post," with its integrated abutment and guide bur, simplifies the clinical procedure and reduces stress on the tooth [18]. Screw posts are increasingly less used due to their excessive rigidity and lower survival rate [19]. Finally, zirconia posts, although rigid and esthetic, cannot be repaired in the event of fracture, which limits their use compared to fiber posts [20].

Materials for assembling post and core restorations

The materials used to assemble post and core restorations vary depending on the type of post and the clinical strategy. In this study, 91.6% of practitioners preferred glass ionomer cement for sealing inlay cores. Glass ionomer cement is valued for its mechanical strength, adhesion to metal posts, fluoride release, and ease of handling. Resin-modified glass ionomer cement (RMGIC) improves these properties, offering better moisture tolerance and superior esthetics.

For bonding fiber posts, 48.5% of practitioners use nonadhesive cements, a result similar to that of a study in Pakistan. Etch-and-rinse adhesive systems provide good adhesion in the coronal area but are less effective in the root area. Nonadhesive cement systems (often universal) containing 10-MDP tolerate moisture better and create a durable chemical bond with hydroxyapatite [20]. In Turkey, 34.1% of practitioners prefer self-adhesive cements. These dual-cure adhesives offer a simple and effective solution for intracanal sealing thanks to their reliable polymerization in a moist environment [21].

Direct post and core restorations

The coronal material provides the prosthetic base for the crown. In the present study, practitioners mostly opted for flowable composite, which was also preferred in studies conducted in India and Pakistan [6]. Its fluidity and wettability promote good cavity adaptation, reducing the risk of displacement while providing a durable esthetic finish [22].

Prognosis and role of posts in post and core restoration

No significant difference in survival has been demonstrated between fiberglass posts and metal inlay cores for endodontically treated teeth without residual walls [23]. Despite their advantageous biomechanical properties, fiber posts do not offer proven clinical superiority. In this survey, 88% of practitioners believe that posts strengthen the tooth, although their role is actually limited to retaining the restoration, with no proven strengthening effect, and may even weaken the root structure [6,14]. 

Limitations

The study presents several types of bias and methodological limitations, primarily related to its scope, the data source, and the nature of the data collection process:

Representativeness (Geographic) Bias

The survey was strictly limited to private-sector dentists within the Casablanca-Anfa prefecture. Consequently, the results obtained cannot be generalized to all practitioners in the city of Casablanca or at the national level.

Selection Bias (Related to the Sampling Frame)

The list of practitioners used for sampling was provided by the regional council. It did not include dentists who had not renewed their annual membership.

Reporting Bias

As this was a cross-sectional epidemiological study using a questionnaire as a tool, the data collected relied solely on the dentists' self-declarations. There may be a discrepancy between the clinical practices actually performed chairside and those that practitioners chose to report in their responses.

Nonresponse Bias

This bias was significantly minimized in this study due to an exceptional participation rate of 99.59% (244 valid responses out of 245 solicited dentists).

Finally, the study encountered difficulties with participant cooperation, as some practitioners required multiple reminders over a period of more than two weeks to complete the questionnaire.

## Conclusions

This study showed that direct post-and-core restorations were the most commonly reported technique among private dentists in the Casablanca-Anfa prefecture, with a high frequency of use (90.2%). Fiberglass posts, nonadhesive cements, and flowable composites were among the most frequently used materials in clinical practice.

Indirect techniques were reported to be used in specific and more complex clinical situations, while the adoption of CAD/CAM inlay-core technologies appeared to remain limited. These findings suggest variability in clinical practices and highlight the potential need for continuing education to support the integration of evolving materials and technologies in post-and-core restorations.

## Figures and Tables

**Figure 1:**
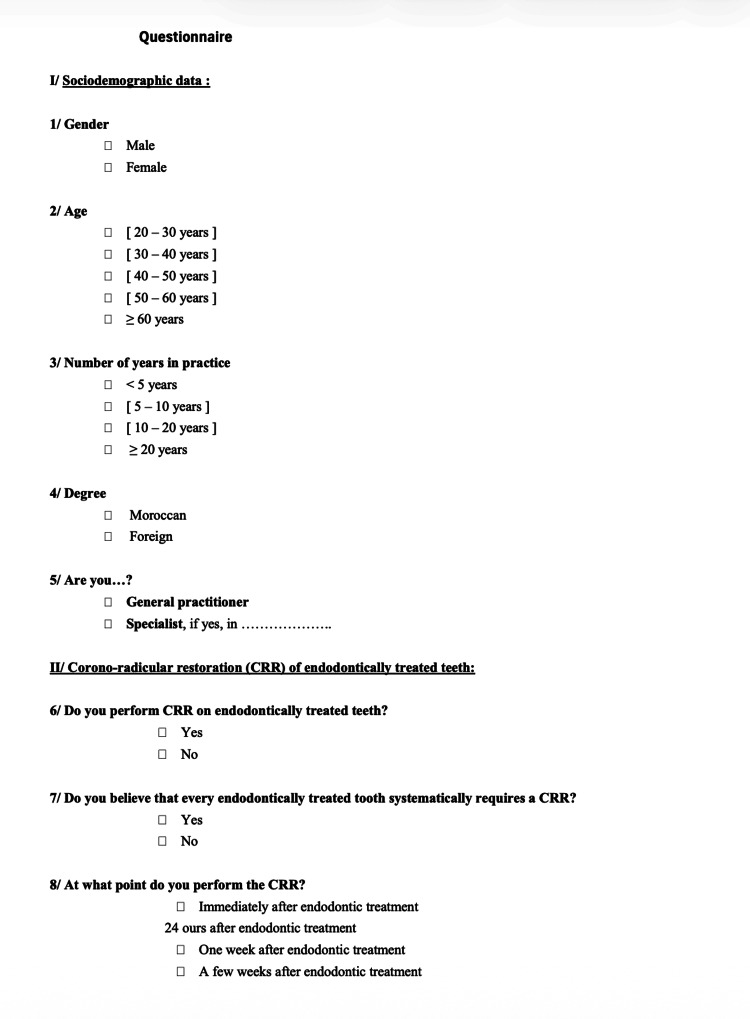
Corono-radicular restorations of endodontically treated teeth: questionnaire used in this study (part 1)

**Figure 2:**
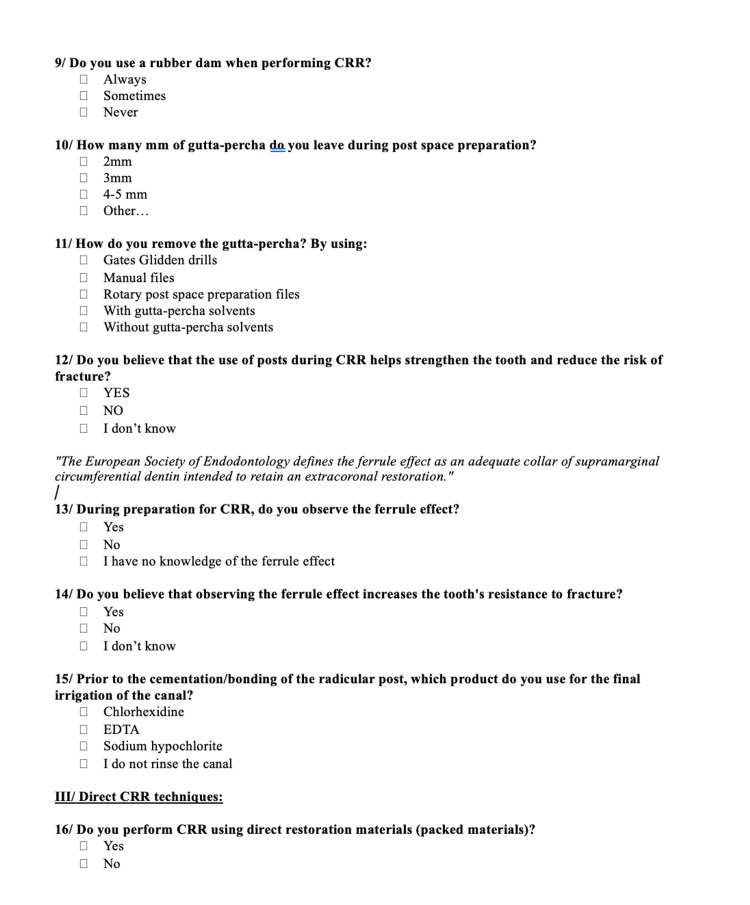
Corono-radicular restorations of endodontically treated teeth: questionnaire used in this study (part 2)

**Figure 3:**
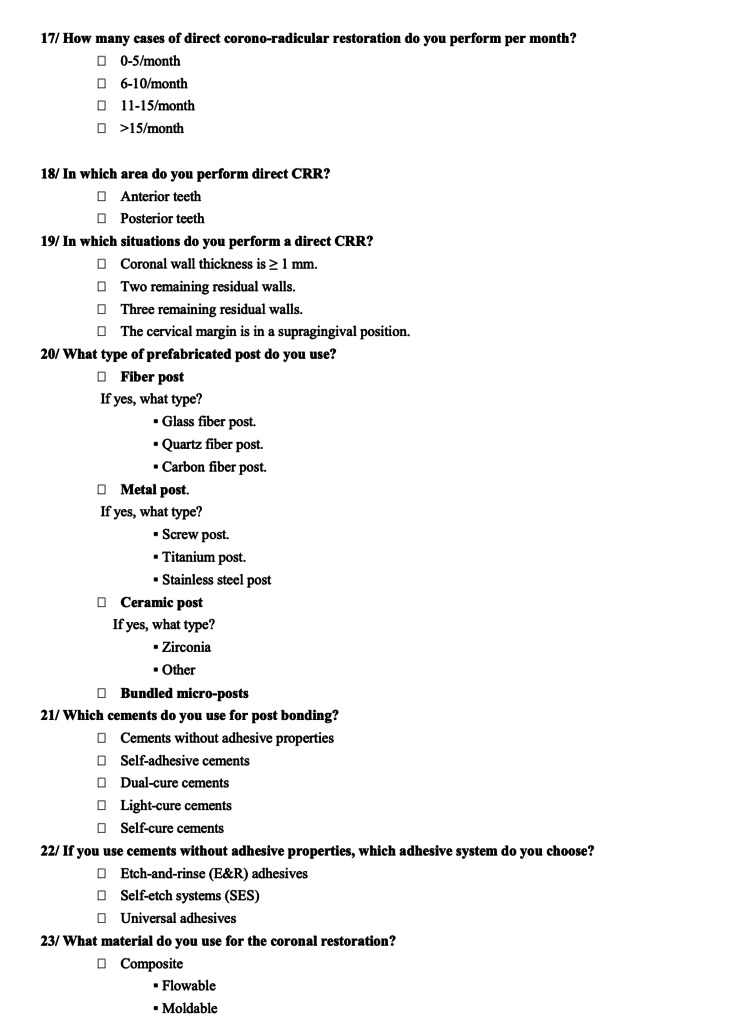
Corono-radicular restorations of endodontically treated teeth: questionnaire used in this study (part 3)

**Figure 4:**
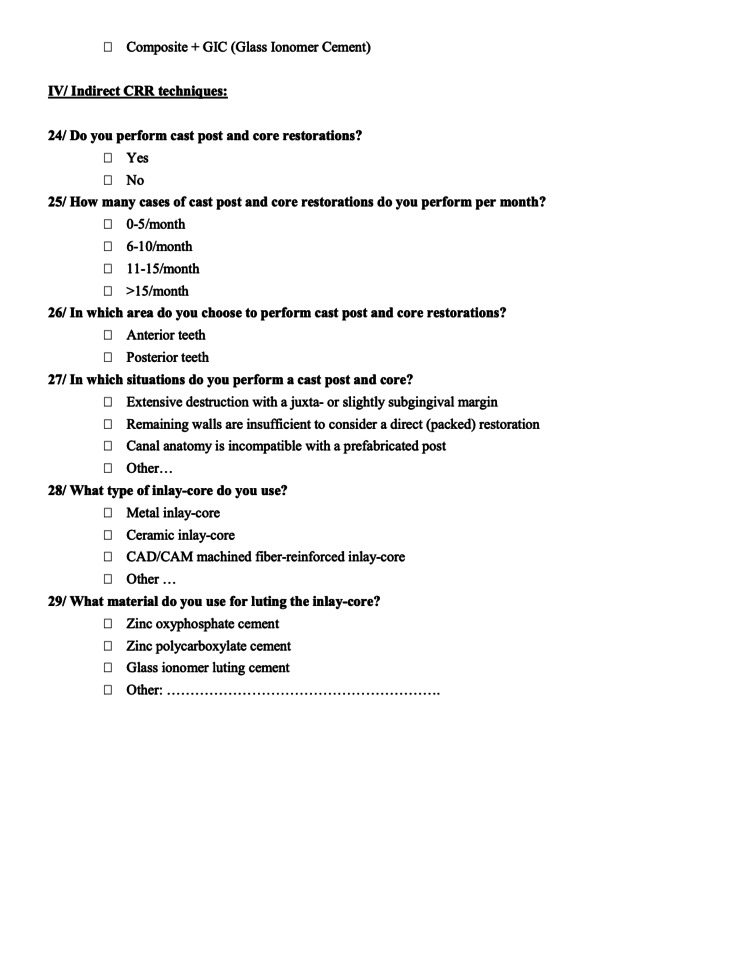
Corono-radicular restorations of endodontically treated teeth: questionnaire used in this study (part 4)
